# Enhancing communication skills for pediatric visits through on-line training using video demonstrations

**DOI:** 10.1186/1472-6920-8-8

**Published:** 2008-02-11

**Authors:** Kathi J Kemper, Jane M Foy, Larry Wissow, Steve Shore

**Affiliations:** 1 Departments of Pediatrics and Public Health Sciences, Wake Forest University School of Medicine, Winston-Salem, NC 27157, USA; 2 Departments of Psychiatry and Pediatrics, Johns Hopkins University, Baltimore, MD, USA; 3 Executive Director, NC Pediatric Society, Raleigh, NC, USA

## Abstract

**Background:**

Training in communication skills for health professionals is important, but there are substantial barriers to individual in-person training for practicing clinicians. We evaluated the feasibility and desirability of on-line training and sought suggestions for future courses.

**Methods:**

Based on successful in-person curricula for communication skills and our previous on-line curricula, we created an on-line course consisting of 28 modules (4.75 hours CME credit) about communication skills during pediatric visits that included a mental health concern; each module included a brief case, a multiple choice question, an explanation, and a 1–2 minute video demonstrating key skills. Specific communication skills included: greeting, setting an agenda, discussing diagnosis and treatment, and managing negative interactions. The course was announced by emails in spring, 2007; the course was available on-line for 60 days; we aimed to enroll 50 clinicians. Outcomes were analyzed for those who evaluated the course within 75 days of its initial availability.

**Results:**

Overall, 61 clinicians registered, of whom most were nurses (N = 24), physicians (N = 22), or psychologists or social workers (N = 12). Of the 36 (59%) clinicians who evaluated the course, over 85% agreed that all course objectives had been met; over 90% reported greater confidence in greetings and agenda-setting; and over 80% reported greater confidence in discussing diagnosis and treatment and managing negative interactions. Nearly all, 97% would recommend the course to other clinicians and trainees. Suggestions for improvement included a library of additional video vignettes and written materials to accompany the on-line training.

**Conclusion:**

On-line training in communication skills for pediatric mental health visits is feasible, desirable and associated with increased confidence in key skills. Positive feedback from clinicians suggests that a comparison of on-line versus in-person training is warranted.

## Background

Up to 20% of children and adolescents in the United States are thought to have an emotional or behavioral disorder, and twice as many children have functional problems related to behavior or feelings, even though they don't meet criteria for a particular diagnosis [1-5]. Half of all primary care office visits involve behavioral, psychosocial or educational concerns [6,7]. Mental disorders are largely chronic conditions, and most begin in childhood or adolescence.

A main strategy for improving children's mental health care has been to position services where children spend their time. This includes increasing the mental health service capacity of primary medical care providers [8-10]. Reflecting the importance of this primary care strategy, the American Academy of Pediatrics, the American Academy of Family Practice, and the National Association of Pediatric Nurse Practitioners haveeach launched educational programs about mental health care. These organizations recognize that primary care clinicians are well positioned to detect emerging mental health issues[11,12].

Although pediatricians are identifying more children with behavioral and mental health problems than they did in the past [13], the vast majority of children with these problems remain undiagnosed and untreated [14,15]; for example, primary care clinicians identify only about a quarter of children and adolescents with mental problems [16,17]. Clinicians' lack of training remains a significant barrier in identifying problems and providing mental health services to pediatric and adolescent patients with psychosocial problems [18-20]. In one study, more than half of parents with emotional, behavioral, or developmental concerns about their children did not discuss them with their child's doctor [17]. Even when cases are detected, problems may be under-treated and receive minimal follow-up [21]. Furthermore, even among physicians who prescribe antidepressant medications, most provide limited information to patients about their medications, resulting in unfilled or non-renewed prescriptions [22].

Clinicians providing mental health care to children and adolescents need communication skills to elicit mental health concerns from those who may be reluctant to disclose them and who may not see that clinician as a source of help. Research has identified common communication patterns that affect clinician-patient discussion of psychosocial problems [20,23,24]. For example the quality of care for depressed patients improves when physicians explore and validate patient concerns [25]. Clinicians need skills to explore the family's perceptions about their problems and their readiness to seek help. If clinicians find the child and/or family unmotivated, embarrassed, resistant, hopeless, angry, conflicted, or otherwise unready to address the issue, they need skills to address these barriers.

Training primary care clinicians to use communications skills applicable to the disclosure and management of emotional problems shows promise as one way of increasing the mental health service capacity of primary care [26-29]. Optimal communication skills enhance patients' trust, satisfaction and adherence with treatment recommendations [30-36]. Communication skills reflecting respect and empathy can be taught, learned and practiced by health professionals [28,34,37-39].

Training in advanced communication skills (readiness to change, conflict management, and dealing with emotionally challenging patients) is generally a high intensity endeavor; typically, it includes a low student-teacher ratio, often using video tape to observe desired behaviors and role plays to practice them [40-45]. While in-person training by a child psychiatrist appears to be effective in enhancing clinician's skills in these areas, it is costly and limited in terms of the number of clinicians who can be trained. Not all clinicians have ready access to in-person training sessions, and skilled trainers may not be available, particularly in rural areas [46,47].

One successful approach to educating diverse, widely dispersed busy clinicians is the use of internet-based curricula [48-50]. As access to high speed internet connections improves, it becomes feasible to imbed brief video vignettes or demonstrations into web-based courses, enhancing education related to skills as well as knowledge [51]. These tools have been used successfully for internet-based or telemedicine courses on surgical procedures [51,52], but they have not been applied to curricula addressing communication skills during mental health-related visits for primary care clinicians.

The aim of this study was to assess the feasibility and desirability of delivering an on-line course on communication skills for pediatric mental health visits using an internet-based platform incorporating brief video demonstrations of desired skills. We defined feasibility as the ability to recruit diverse health professionals to the on-line course with few technical barriers reported by enrollees; we defined desirability as positive feedback about the course from enrollees. We were also interested in obtaining enrollee suggestions to enhance future courses. These data are essential before embarking on costly studies comparing the effectiveness of standard in-person training to on-line training to enhance communication skills for clinicians engaged in mental health care of children.

## Methods

This was an evaluation of an educational program provided over the internet during the spring of 2007.

### Subjects and Recruitment

Subjects were eligible to enroll if they were a licensed health care provider. We aimed to enroll a convenience sample of 50 clinicians with some expertise in pediatric mental health. Recruitment was done via email from the authors to five groups of pediatric primary care clinicians and pediatric mental health professionals (physicians, nurses, psychologists and social workers): the Multidisciplinary Mental Health/School Health Committee of the North Carolina Pediatric Society (N = 57), the Community Care of North Carolina Behavioral Health Integration Pilot sites (N = 64), nurses in the Child and Family Service Teams in Forsyth County, NC (N = 7), the American Academy of Pediatrics Mental Health Task Force (N = 220), and School Health Alliance for Forsyth County staff (N = 10). Two emails (sent April 12 and May 1) were sent to each of these groups; these emails may have been forwarded. Emails were also sent to the two pediatric chief residents at Wake Forest University School of Medicine (WFUSM). The emails invited participants to help "evaluate" and "beta test" the curriculum. Although the program was offered through the Northwestern North Carolina Area Health Education Center's web site (NWAHEC), it was not actively marketed by NWAHEC. Furthermore, although continuing education credit was offered through the WFUSM Office of Continuing Medical Education (CME), the course was not advertised through the CME or alumni offices because the primary purpose was to collect feedback about the program.

### Course content

The course content was based on an in-person training funded by the National Institutes of Mental Health (RO1 MH62469). The in-person training used a case-based interactive, one-to-one teaching approach including written materials; video taped illustrative vignettes; role playing and feedback on one-to-one feedback on trainees' videotapes with standardized patients (See Appendix for example of one section of one module). The in-person training required approximately 4 hours of learner time, including 3 one hour individual or small group sessions with a child psychiatrist and the standardized patient.

The curriculum included material relevant to eliciting parent and child mental health concerns, developing an agenda for the visit, prioritizing concerns, partnering with families to develop an acceptable treatment plan, and increasing expectations that treatment would be helpful. The course materials were consistent with principles of patient-centered care and relied on motivational interviewing tools such as identifying barriers to change and rolling with resistance. Materials were arranged by learning objectives; a content outline was developed based on these objectives. Both the objectives and content outline were reviewed for accuracy and completeness and modified as needed. Based on the outline, we divided the objectives and content into eight (8) "sections" comprising the overall course. Each section built on prior sections. The sections were further divided one or more modules to make each learning section easier to complete on-line (Table 1).

**Table 1:** Course Sections, number of modules and amount of CME credit per section

**Section**	**Number of Modules**	**CME credit in hours**	**Number video demonstrations**	**Number of Enrollees who completed each Section for CE/CME Credit**
Goals for Pediatric MH Visit	4	0.5	3	35
Epidemiology and Description of Pediatric MH Problems	4	0.5	0	26
Barriers to MH Care for Youth	1	0.25	0	25
Greetings	4	0.5	4	23
Agenda-Setting	5	0.5	5	19
Discussing Diagnosis	1	0.25	2	18
Developing Treatment Plans/Giving Advice	1	0.25	2	18
Managing Negative Interactions	8	2.0	12	15

**TOTAL**	**28**	**4.75**	**28**	**36 completed formal course evaluations**

### Course organization

The organization and format of the curriculum were based on earlier studies on internet-based education funded by NIH NLM (R01 LM007709) [48,50]. Each module had a learning objective; a brief outpatient clinical scenario featuring a parent and child with a mental health concern; a teaching question; the "preferred" answer with a 1–3 page explanation; if appropriate, one or more 1 to 2 minute video(s) illustrating the desired communication behavior (28 videos altogether); and, if appropriate, resources and tools to assist patients with behavior change. Most of the videos had been used in the in-person training and featured one of the co-authors; additional videos were made specifically for this course to cover gaps. In the on-line module, an image of one screen of the video was located at the bottom of the explanation; learners simply clicked on the image to link to the video file.

After the content was thoroughly reviewed by the co-authors and modified as needed, a post-test was created for each section. Programming to put the materials on-line was done by NW AHEC staff, reviewed and modified as needed. Free Continuing Medical Education (CME) credit was provided for each section for participants who scored 70% or higher on the section post-test.

### Course evaluation and feedback

At the close of the course (two weeks after enrolling the target of 50 clinicians), we asked participants by email to complete the on-line course evaluation, even if they had not completed all of the course sections or received any CME credit. Two reminder emails were sent. Complete web-based evaluations received within 10 weeks of placing the course on-line were included in this analysis. Brief email communications about the course (e.g. "Great, I really enjoyed it!") sent in response to the email requests to complete the on-line evaluation were not included in the formal analysis.

All data were entered on-line and analyzed anonymously using simple descriptive statistics.

The Wake Forest University Health Sciences Institutional Review Board approved this project as educational research.

## Results

Although the recruitment goal was 50 participants, 64 health professionals enrolled by 5/16/07. Of these, 95% were from North Carolina; others were from nearby states and one was from Germany. Most (81%) were women. Data on age and race were not collected. Nearly all were the targeted professional groups: 24 nurses (including nurse practitioners and clinical nurse specialists), 22 physicians, and 12 social workers or psychologists; the other participants were mental health counselors or administrators.

Enrollees tended to study the course sections in the order presented (Table 1). By May 30, 2007 when the course was closed, over half of the participants (N = 35) had completed the CME questions for the first section. Fewer completed CME questions for each of the subsequent sections: Epidemiology of Pediatric Mental Health (MS) Problems (26), Barriers to MH Care for Youth (25), Greetings (23), Agenda-Setting (19), Discussing Diagnosis (18), Developing Treatment Plans/Giving Advice (18), and Managing Negative Interactions (15). No enrollees reported dropping the course for technical reasons. Several reported that they felt able to provide meaningful feedback based on reviewing just a few modules. Completion rates for the CME questions for all eight sections were highest for psychologists and social workers (5/12 who enrolled), next highest for nurses (7/24), lower for physicians (3/22) and lowest (0) for the others. Data on gender, age and other demographic characteristics of completers versus non-completers of the CME questions are not available.

As expected from the recruitment strategy, enrollees were experienced in providing care to pediatric patients with mental health concerns Among the 36 (59%) participants who provided formal course feedback, 32 (89%) reported having seen a pediatric outpatient in the previous month; 76% of these 32 reported discussing a mental health concern in more than 10% of their clinic visits (23%) reported discussing a mental health concern with more than 90% of patients).

Overall feedback about the course was very positive. Nearly all (97%) of those who gave feedback (and all four of those who did not respond to the formal review but did send emails later) said they would recommend the course to others. Most agreed that the course had met its objectives (Table 2). Specifically, 96% agreed or strongly agreed that the course helped them better identify and clarify mental health needs and use a collaborative style of communication; and 94% agreed or strongly agreed that the course helped them better manage negative interactions during pediatric mental health visits, such as a rambling, angry, demoralized or conflicted family or patient. The percentage of respondents who agreed that they were more confident in specific skills after the course than before taking it ranged from 80% (for discussing a diagnosis) to 90% (for greeting and setting an agenda); 83% reported they were more confident managing negative interactions and 86% said they were more confident discussing treatment.

**Table 2:** Participants agreeing that specific course objectives had been met

**Course Objectives**	**Percent strongly agreeing or agreeing objective met (N = 36)**
Support family confidence and hope	97%
Identify and clarify mental health needs during visit	96%
Use collaborative, style of communication	96%
Facilitate and build agreement on Diagnosis, Evaluation and Treatment	94%
Manage negative interactions (visits with demoralized, angry, embarrassed, rambling, disagreeable pt or family)	94%
Reduce family/child conflict	93%
Enhance confidence in caring for children with MH problem	93%
Enhance confidence in diagnostic info/advice accepted	85%

Feedback about the structure and format of the course was also positive, with 94% agreeing that the course used effective teaching methods. Most respondents (83%) felt the number of sections (8) was "about right," with the remaining respondents split between those who wanted more and those who wanted fewer. The amount of time allocated for CME (4.75 hours total) reflected the amount of time most participants spent on the course; 55% of completers spent 3–4 hours, and 14% spent 5 or more hours. Most liked the course organization, noting that the "Modules flowed well and (were) built on previous content – clear and easy to follow"; participants also liked the opportunity to "do it at my own pace."

We were particularly interested in feedback about the video demonstrations. Most (72%) respondents felt the course would not be as good without the videos. Most (64%) also felt the number of videos (28) was "about right," with most of the remainder (28%) indicating that they would have preferred fewer videos, noting that there was some redundancy, particularly in the latter sessions. This finding was supported in the number of video clips actually viewed: 90% or more of the videos were watched by only 40% of those who completed the CME questions for all sections. A few participants requested more videos, suggesting a repository of additional examples of challenging situations showing diverse (age/gender) clinicians and patients as an option for learners who desired more demonstrations. The vast majority of participants accessed the content including the videos without difficulty, though some complained that without a high speed internet connection, they did not like waiting for the videos to load.

In response to the questions of what they gained and how they intended to change their practice based on the course, participants provided qualitative feedback for most, but not all sections. None commented on what they'd learned or planned to change as a result of the sections on epidemiology or barriers to care. However, they reported greater confidence over all in addressing mental health issues: "less afraid to approach mental health issues" and "willing to address mental health issues more readily, collaborate better with family." Also, participants felt more confident about basic communication skills during visits: "Setting the tone in a mental health visit more appropriately," "how I introduce myself", "the way I go about setting an agenda," "more collaborative interactions," and "I will be more comfortable working with patients/families to set an agenda even if it means asking them to return to address topics we couldn't adequately cover in a single visit". They also reported gaining skills in reflective listening: "Restating what patient or parent have given as reason for appointment and asking them questions to expand that" and patient/family centered, collaborative care: "Build consensus toward plan of care" and "Involve family more in developing treatment plan."

The largest section of the course, illustrating the most demanding skills, was on managing negative interactions. This section also engendered several comments about what participants had learned and what they planned to do. For example, "I plan to utilize some of the worksheets and other web sites in having a plan when I deal with these issues (managing negative interactions)"; I plan to "use info about managing negative interactions, use pros and cons and other work sheets." Some specifically learned from the specific approach skills related to changing behavior; for example, one respondent reported plans to use "more reframing and helping to set measurable goals".

Participants also provided comments on what they liked most and what changes they would suggest to the course. Most liked the case scenarios, the concise practical explanations and the videos; one participant specifically commented that these situations are "just like my practice." Of those who completed the CME questions for all eight sections of the course, the favorite section was "Managing Negative Interactions." When asked what changes they would make to the curriculum, most respondents said "none" or left the question blank. Several requested written materials, a summary, or a workbook to accompany the on-line materials to use as a reference. Another wanted more video vignettes with variations in a repository. Several wanted the opportunity to review or update the materials regularly so as to better incorporate some of the behavioral changes. A few suggested minor wording changes to case questions or CME test questions. One participant wanted to do more work with children with mental health problems and requested additional on-line training in how to provide psychotherapy.

## Discussion

These data demonstrate that it is feasible and desirable to provide an on-line course including brief video demonstrations of communication skills during pediatric visits that include mental health concerns. Response rates to on-line requests are typically approximately 1%; for the 300+ emails we sent, over 60 persons enrolled. Although the enrollment period was brief, enrollment exceeded the recruitment goal by 28%, and the course drew diverse clinicians (physicians, nurses, psychologists, social workers and others). The only technical problems related to the speed of downloading video vignettes for those that did not have high speed internet connections; none dropped out due to technical problems. The on-line course also appeared to be desirable. Feedback was positive, and the vast majority, even those who did not complete the CME questions for all the sections of the course, would recommend it to others.

Compared with in-person training, computer-based and internet-based training has shown comparable benefits for learners' knowledge; furthermore, over time, on-line training may become even more feasible and desirable. For example, in a randomized, controlled comparison, Davis, et al, found similar improvements in knowledge and attitude about evidence-based medicine for an in-person and computer-based course for postgraduate medical trainees [53]; computer-based learning has also proven effective for patient education, enhancing families' safety behaviors and adolescent physical activity, for example [54-56]. Using video clips, on-line training can improve learners' confidence and skills as well as knowledge [57]. As the next generation, which has grown up with the internet and internet-based learning (including undergraduate on-line courses offered through Blackboard^®^, and other providers), joins the health care professions, it is likely that on-line training will become even more desirable [58]. As internet connection and processing speeds continue to improve and as access increases, on-line training becomes even more feasible.

The course feedback provides valuable guidance for future on-line courses. For example, for courses geared toward clinicians who routinely provide mental health care, the sections on epidemiology of mental health problems and barriers to care could be omitted. On the other hand, if offered as a requirement to students or trainees who are less familiar with the clinical challenges and the need for services, these sections might well be included. For all learners, the course might be divided into one hour segments to allow learners to focus on the skills of most interest to them (e.g., developing an agenda versus discussing diagnosis and treatment versus managing negative interactions), to promote engagement and to encourage completion. Each smaller class might include reminders and deadlines to encourage completion. Furthermore, to sustain interest and engagement and reduce the burden on participants with slower internet connections, the number of video clips could be reduced, with others available through an on-line course resource area. The separate resource area could provide multiple video vignettes of the same basic skills demonstrated by different types of clinicians (age, race, gender, profession) as well as additional tools to facilitate communication and behavior change. Future courses should also provide written materials such as workbooks and study tools to accompany the on-line materials.

As a pilot project, this study had several limitations. First, the sample, although larger than anticipated, was still relatively small and self-selected. The short time frame may have excluded some potential enrollees. The self-selected sample makes it difficult to generalize conclusions to clinicians who are less interested in mental health issues. Future studies will need to enroll a larger and more diverse sample. Future courses will also need to create additional videos showing more diverse clinicians demonstrating the desired communication skills. Cost data were not collected; studies comparing the feasibility and costs of on-line versus in-person training will need to include such data. Furthermore, the follow-up period was quite brief and limited to self-report; physicians have a limited ability to self assess accurately [59]. Although self-reported confidence is associated with better identification of mental health problems [2], future studies should include observations of actual clinical behavior. Drop off in completing the CME questions was moderately high; because we did not specifically query non-completers, we are unsure of their reasons for not completing all of the CME questions and course evaluation questions. One possible reason is that the course was marketed as a beta-test soliciting feedback rather than as a formal curriculum. This conclusion is supported by the fact that 36 enrollees provided formal web-based feedback while only 15 completed the CME questions for all 8 sections; furthermore, several additional enrollees provided unstructured email feedback not included in the formal analysis here, suggesting that even more enrollees reviewed the material, but chose not to complete the CME questions or the formal on-line evaluation. The lower evaluation rate by physicians and nurses compared with psychologists and social workers further limits generalizability and suggests the need for more focused evaluation in different professional groups. It is possible that completion rates are hampered by factors in the course itself, its format, its length or its content or some factor in the participants or the short time period allowed for completion. Finally, this course concentrated on communication skills for mental health visits; future research might test feasibility and desirability of such courses for other challenging clinical communication settings such as patients with obesity, sexually transmitted diseases, domestic violence or child abuse.

## Conclusion

Despite these limitations, these data provide a small but valuable first step for educators interested in improving clinicians' communication skills during pediatric visits that include a mental health concern. Although extensive in-person training is the standard, an on-line course on this topic is feasible and desirable. Additional research is needed to evaluate more objective outcomes (actual skills practiced, patient satisfaction, and clinical outcomes), to compare the costs and effectiveness of in-person versus on-line training, and to explore the feasibility of this format for other challenging communication settings in clinical practice.

## Competing interests

The author(s) declare that they have no competing interests.

## Authors' contributions

KK conceived of the on-line training, wrote all the modules, analyzed the data and drafted the manuscript.

JF conceived of the project, reviewed all modules, recruited participants and participated in revising the manuscript.

LW developed the in-person training on which the on-line curriculum is based, reviewed and revised the on-line content, and participated in drafting and revising the manuscript.

SS helped create video vignettes for the course, disseminated information about it to members of the NC Pediatric Society and participated in revising the manuscript.

All the authors have read and approved the final version of this manuscript.

## Appendix – Example of a section within a module

### Objective

By the end of the section on "Greetings", participants will be able confidently to greet patients and their family members/guardians appropriately. Specifically, an appropriate greeting includes:

• Tone of voice: warm and respectful

• Names: greet each person in the room by name

• Words: "I want to hear from all of you; who wants to go first?"

• Non-verbal behavior: handshake or gentle touch for each person, eye contact, posture facing speaker

### Teaching Question for objective 4

*Case*: You are seeing a 16 year old girl, Nancy Verhoeff, who is brought to clinic by her mother, Ms. Brenda Verhoeff, for evaluation of chronic fatigue.

*Question*: While warmly and respectfully greeting Nancy and Ms. Verhoeff by name and asking which of them wants to go first in sharing their concerns, the best non-verbal behavior is to:

a. Efficiently review Nancy's chart while asking about the duration and severity of symptoms as well as precipitating and relieving factors.

b. Turn toward and keep looking at Nancy while you're talking so that Ms. Verhoeff gets the idea that Nancy is the primary concern and Nancy should do the talking now that she's 16 years old.

**c. **Shake hands with Ms. Verhoeff, but avoid touching Nancy until you start the physical examination to avoid touching that might be misinterpreted as sexual.

**d. **Shake hands with both Nancy and Ms. Verhoeff as you greet them. Turn toward whoever is speaking or the person to whom you are speaking; maintain eye contact with whomever is speaking or the person to whom you are speaking.

### Preferred answer

*Answer*: **d is the best answer. **Shake hands with both Nancy and Ms. Verhoef as you greet them. Turn toward whoever is speaking or the person to whom you are speaking; maintain eye contact with whomever is speaking or the person to whom you are speaking.

Explanation:

People who are stressed are extremely attentive to subtleties in body language. Non-verbal behavior is an important way to convey respect and caring.

Clinicians should shake hands with adults and older children and lightly touch younger children on the arm or shoulder. In some cultures, it is considered inappropriate to touch a person on the head, so it is prudent to avoid touching children or infants on the head until you are familiar with the family and their customs and beliefs.

Clinicians should avoid staring at their clipboard (as in answer "a"), computer screen, the medical record, the clock on the wall or the floor while interacting with patients.

Instead, maintain eye contact with the speaker or the person to whom you are speaking. Again, there are cultural differences and distinctions must be made between respectful eye contact and staring.

Generally, if a clinician has a respectful and caring intention, his or her body language will reflect that intention. Similarly, if a clinician is distracted, thinking about a previous patient or a list of things to do, the lack of attention will be conveyed by body language.

Clinicians should ensure that their non-verbal behavior, like their words and tone of voice, reflect a respectful, patient-centered, calm, professional attitude of wanting to serve the best health interests of the patient.

Click < link not included in manuscript > to watch a video of a clinician using respectful non-verbal behavior.

Observe the clinician's behavior.

Reflect on what you observe. What do you think is done well or could be improved?

Click < link not included in manuscript > to find out what others say:

Please practice shaking hands or gently touching each person in the room in a culturally sensitive way. And practice maintaining eye contact.

Reflect, how long do you usually maintain eye contact before starting to look at elsewhere (e.g., notes, the clock, physical characteristics of the patient).

Consider practicing with a colleague or willing patient while being videotaped.

## Pre-publication history

The pre-publication history for this paper can be accessed here:


